# Supplementary material for: Bimodal seismicity in the Himalaya controlled by fault friction and geometry
Source: Nat Commun. 2019 Jan 3;10:48. doi: 10.1038/s41467-018-07874-8 (PMC6318329; doi:10.1038/s41467-018-07874-8)
Supplement: Supplementary file 1 — Supplementary Information [file 41467_2018_7874_MOESM1_ESM.pdf]

Supplementary Information to

Bimodal seismicity in the Himalaya controlled by fault friction and geometry

Luca Dal Zilio[1,*], Ylona van Dinther[2,3], Taras V. Gerya[1] & Jean-Philippe Avouac[4]

[1] Geophysical Fluid Dynamics, Institute of Geophysics, ETH Zürich,
Sonneggstrasse 5, 8092 Zürich, Switzerland
[2] Seismology and Wave Physics, Institute of Geophysics, ETH Zürich,
Sonneggstrasse 5, 8092 Zürich, Switzerland
[3] Department of Earth Sciences, Utrecht University,
Budapestlaan 6, 3584 CD, The Netherlands
[4] Geological and Planetary Sciences, California Institute of Technology,
Pasadena, California 91125, USA

* Correspondence and requests for materials should be addressed to *luca.dalzilio@erdw.ethz.ch*.

Material	*Flow law*	ρ_0 ($kg\ m^{-3}$)	E_a ($kJ\ mol^{-1}$)	V_a (J/bar)	n	η_0 ($Pa^n\ s$)	Hr ($\mu W m^{-3}$)	G (GPa)	μ_s
Fault rock (MHT–fault)	Wet Qz[a]	2750[b]	154[b]	0.8[b]	2.3[b]	1.97 x 10^{17} [b]	2.5[a]	20[k]	0.06–0.2[f,i,j]
Sediments (Ganga Plain)	Wet Qz[b]	2600[h]	154[b]	1.2[b]	2.3[b]	1.97 x 10^{17} [b]	2.25[a,e]	25[g,k]	0.35
Metasediments (Himalayan wedge)	Wet Black Hills Qz[c]	2700[c]	223[c]	0.8[b]	2.3[b]	1.97 x 10^{17} [b]	2.2[f]	25[g,k]	0.45[l]
Tethyan sediments (Tibetan Plateau)	Wet Qz[b]	2750[b,e]	154[b]	0.8[b]	2.3[b]	1.97 x 10^{17} [b]	1.0[a,e]	25[g,k]	0.50[m]
Upper cont. crust (India)	Granite[d]	2670[e]	190[c]	0.8[b]	3.3[c]	1.97 x 10^{17} [b]	2.25[a,e]	25[g,k]	0.60[m]
Lower cont. crust (India)	Diabase[d]	2900[d,e]	276[c]	0.8[b]	3.0[c]	1.25 x 10^{21} [b]	0.4[a,e]	25[g,k]	0.60[m]
Upper cont. crust (Eurasia)	Wet Qz[b]	2670[e]	154[b]	0.8[b]	2.3[b]	1.97 x 10^{17} [b]	1.00[a,e]	25[g,k]	0.50[m]
Lower cont. crust (Eurasia)	Dry–Maryland Diabase[c]	2775[e]	238[c]	0.8[b]	4.7[b]	4.8 x 10^{22} [b]	1.50[a,e]	25[g,k]	0.60[n]
Mantle (India/Eurasia)	Olivine[b,d]	3300[b,h]	532[b]	0.8[b]	3.5[b]	3.98 x 10^{16} [b]	0.0022[h]	67[g,k]	0.60[n]

Supplementary Table 1: Thermo-mechanical visco-elasto-plastic parameters and flow laws corresponding to the adopted lithologies are based on a range of laboratory experiments, previously used data sets, and geological and geophysical constraints. ρ_0 is the reference density, E_a is the activation energy, V_a is the activation volume, n is the stress exponent, η_0 is the reference viscosity, Hr is the radiogenic heat production, G is the shear modulus, and μ_s is the static friction coefficient. Other properties for all rock types: cohesion C = 10 MPa [i], specific heat capacity C_p = 1000 J kg^{-1} K^{-1}, thermal conductivity K = 2.5 $Wm^{-1}K^{-1}$ [a], thermal diffusivity κ = 10^{-6} m^2 s^{-2} [a]; thermal expansion α_ρ= 2.4x10^{-5} K^{-1} [a] and compressibility β_ρ=2x10^{-11} MPa^{-1} [a].

References: *a*) [1]; *b*) [2]; *c*) [3]; *d*) [4]; *e*) [5]; *f*) [6]; *g*) [7]; *h*) [8]; *i*) [9]; *j*) [10]; *k*) [11]; *l*) [12]; *m*) [13]; *n*) [14].

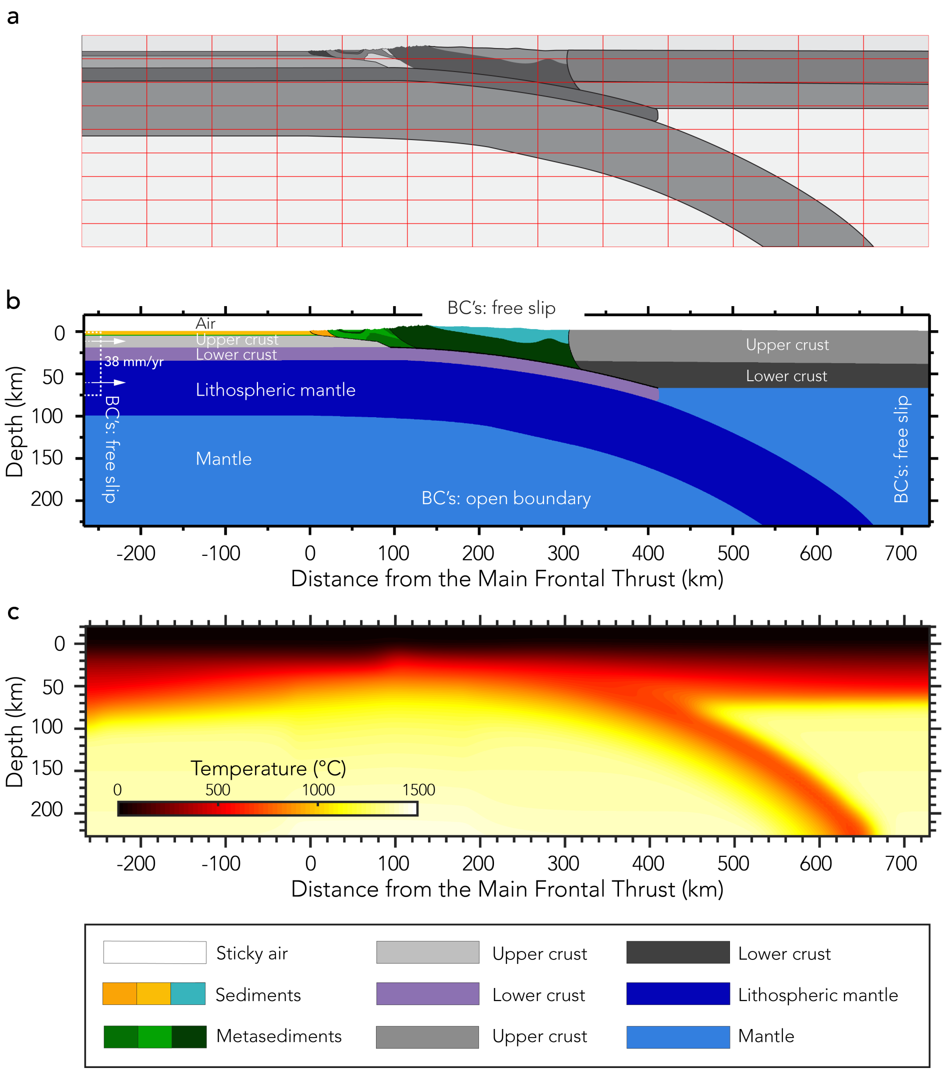

Supplementary Figure 1: Initial model configuration, depicting the (**a**) initial geometry, (**b**) composition including mechanical boundary conditions, and (**c**) temperature distribution for the entire modelling domain. Horizontal distance is from the Main Frontal Thrust. Bottom figurations clarify different rock types.

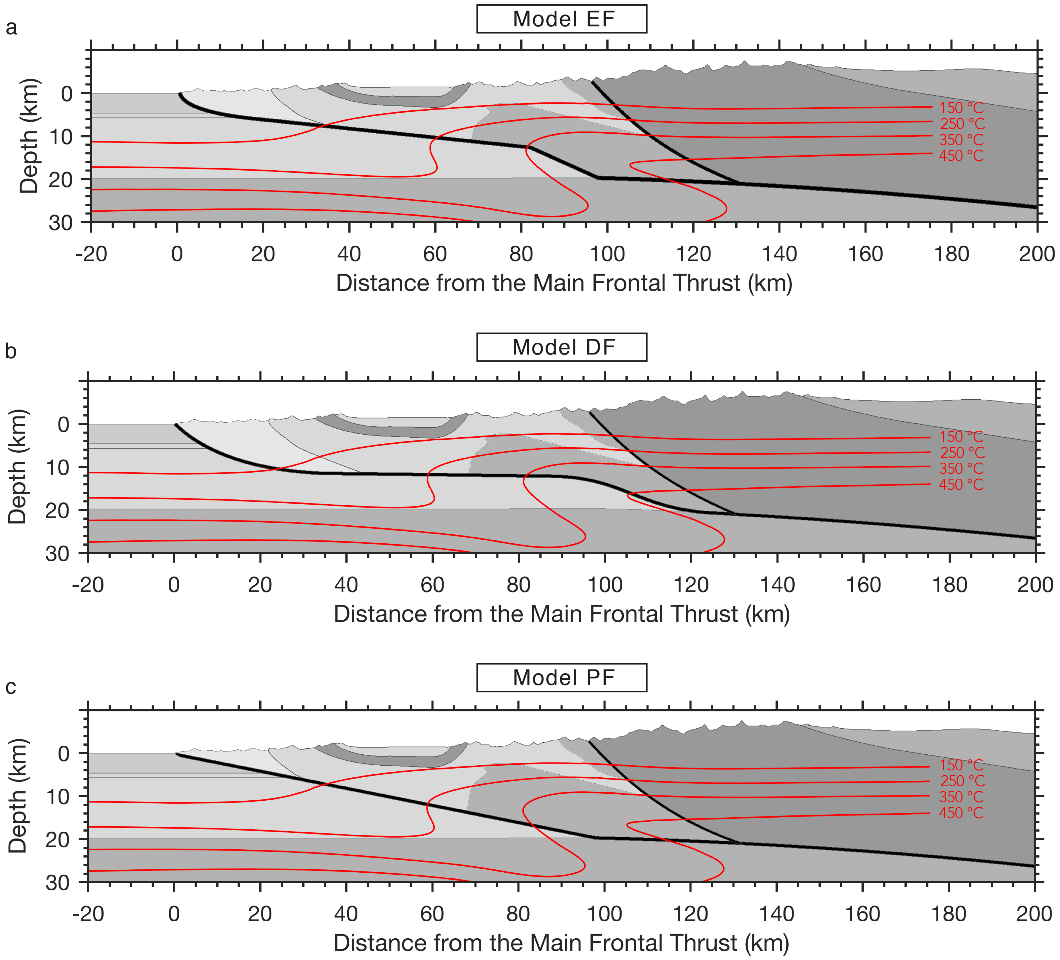

Supplementary Figure 2: The three geometries of the MHT employed in the numerical experiments. Panel (**a**) and (**b**) display the realistic MHT geometry inferred from Elliott *et al.* (ref. 15, Model EF) and Duputel *et al.* (ref. 16, Model DF), respectively, whereas model setup in (**c**) utilises a planar fault geometry (Model PF).

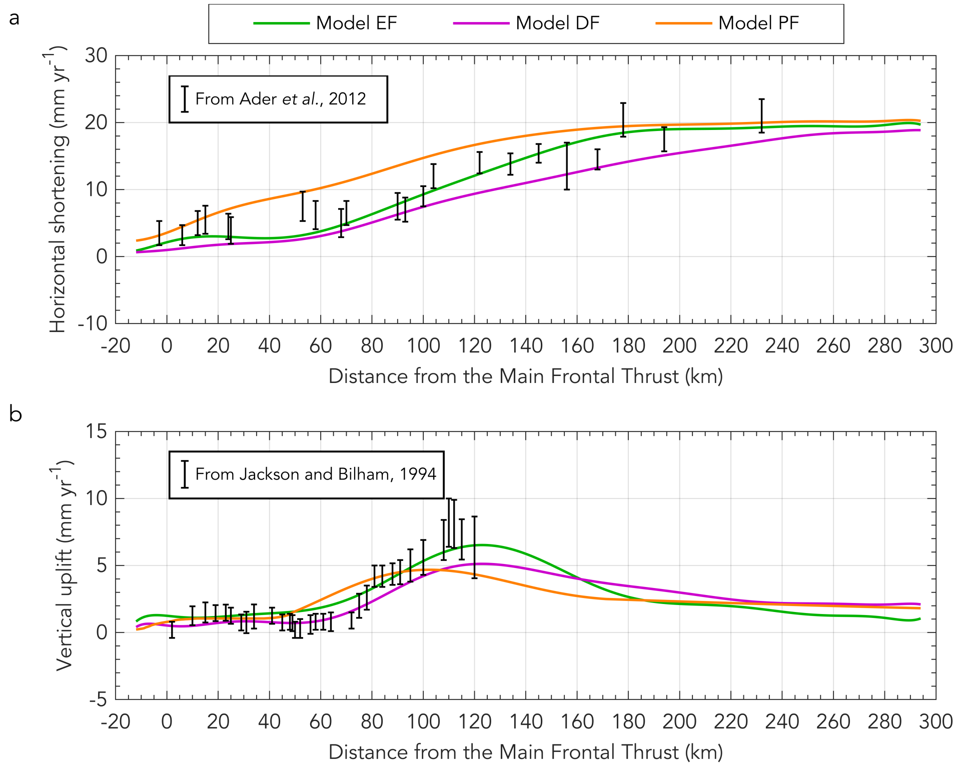

Supplementary Figure 3: Observed and synthetic present-day velocity fields for the Himalaya and southern Tibetan plateau. Black error bars indicate the horizontal and vertical observed fields. Synthetic fields are shown in orange, green and blue for the three MHT geometries employed. The models are driven by a background convergence rate of 38 mm yr^{-1} (ref. 17).

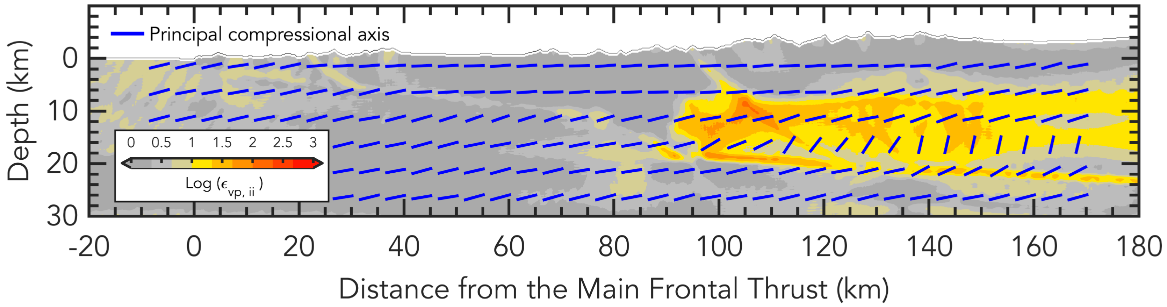

Supplementary Figure 4: Visco-plastic component of strain computed over the long-term interseismic deformation. It shows a sub-horizontal shear zone that closely follows the midcrustal reflector imaged by the INDEPTH experiment[18].

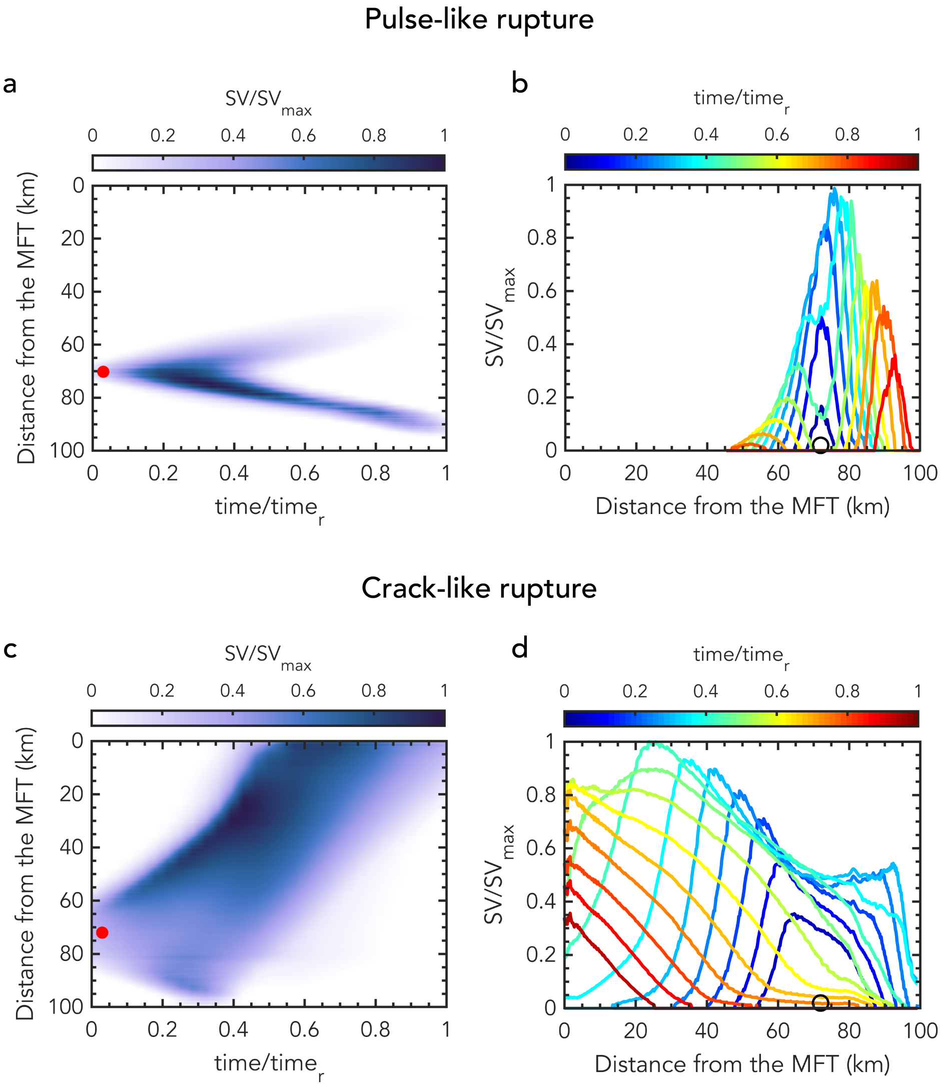

Supplementary Figure 5: Examples of pulse-like (a,b) and crack-like ruptures styles (c,d) along the downdip width of the MHT (0–100 km). To compare the rupture styles, the time is scaled by the total rupture time (time$_r$), whereas the slip velocity is scaled by the maximum slip velocity (SV$_{max}$). Evolution of slip velocity through time (left column, panels **a** and **c**) and along the fault (right column, panels **b** and **d**). Full circle indicates the hypocenter. Pulse-like rupture is characterised by a significant self-healing during the rupture propagation. In contrast, a crack-like rupture shows a continuous slip, almost until the end of the rupture.

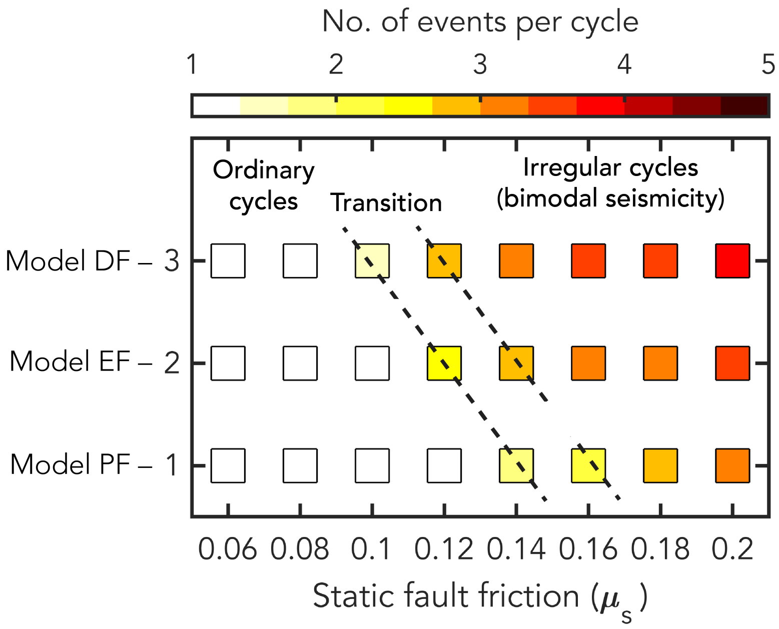

Supplementary Figure 6: Influence of static fault friction and geometry of the MHT on the number of events per cycle. Dashed line roughly indicates the transition between ordinary cycles and irregular cycles. Black frames indicate reference models and figures.

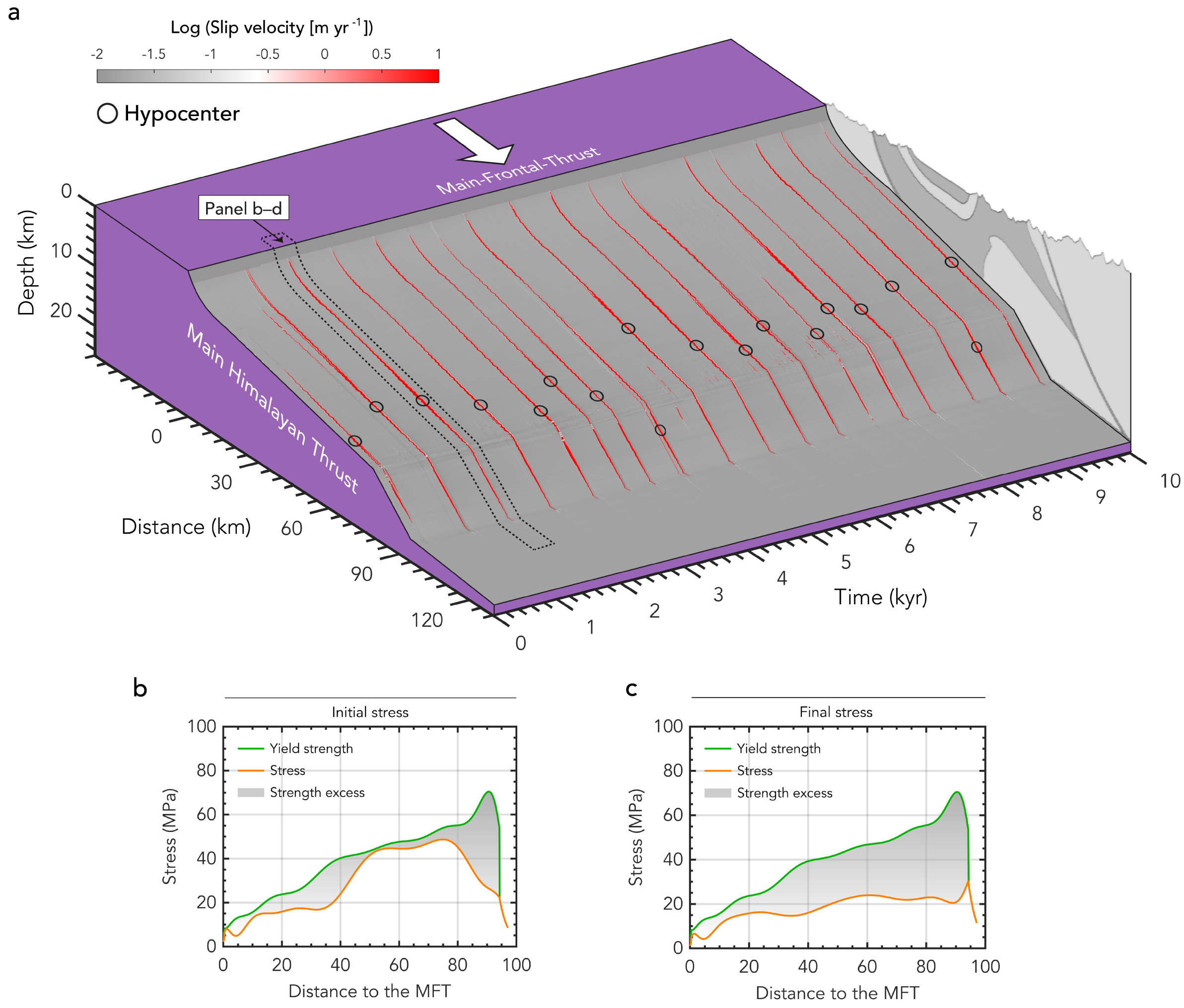

Supplementary Figure 7: MHT–fault behaviour adopting a lower fault friction (μ_s=0.1). **a**, Spatiotemporal evolution of slip on the MHT. Red lines show slip during the simulated earthquakes. **b-c**, Along interface profiles of (**b**) initial and (**c**) final second invariant of the deviatoric stress tensor and strength of the event highlighted in (**a**).

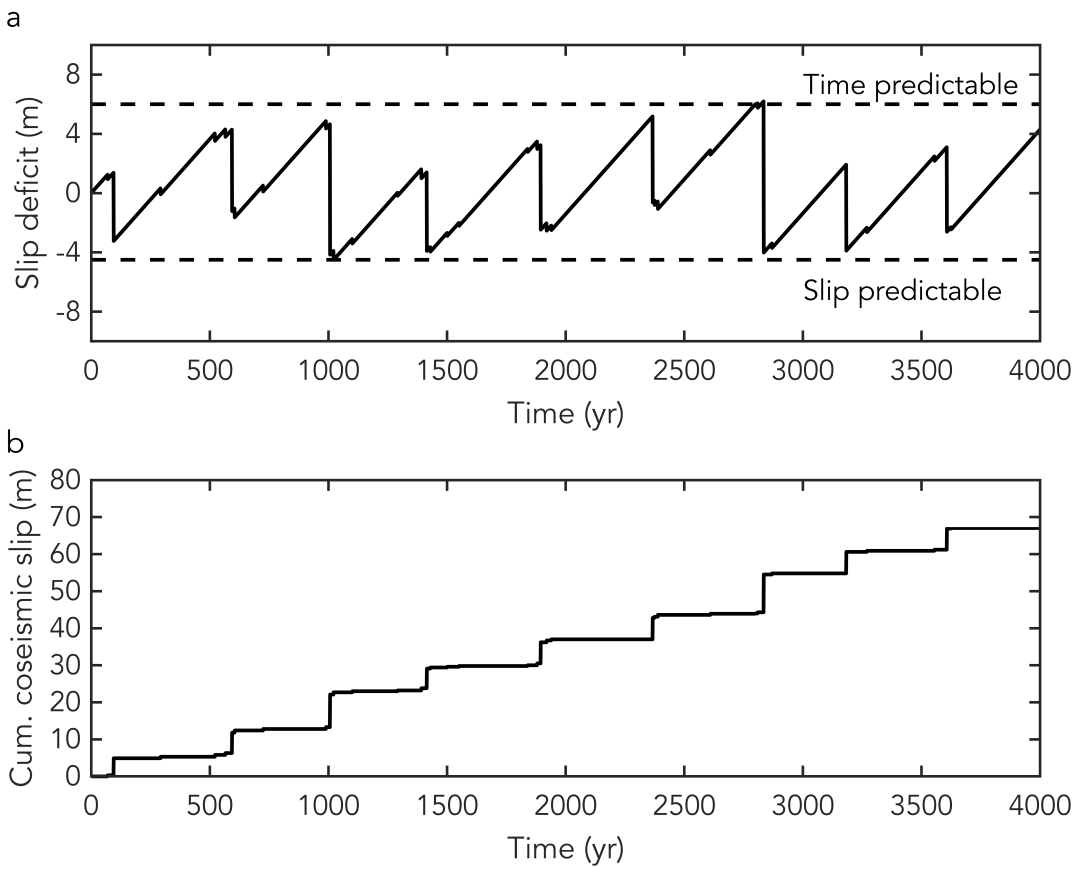

Supplementary Figure 8: An example of long-term fault behaviour computed in the 2D model (Model EF shown in Fig. 3). **a**, Time evolution of slip deficit of the MHT. Dashed lines illustrate that the model is neither time nor slip predictable. **b**, Cumulative coseismic slip against time.

SUPPLEMENTARY REFERENCES

1. Bollinger, L., Henry, P. & Avouac, J. Mountain building in the nepal himalaya: Thermal and kinematic model. *Earth and Planetary Science Letters* **244**, 58–71 (2006).

2. Ranalli, G. *Rheology of the Earth* (Springer, 1995).

3. Gleason, G. C. & Tullis, J. A flow law for dislocation creep of quartz aggregates determined with the molten salt cell. *Tectonophysics* **247**, 1–23 (1995).

4. Burg, J.-P. & Podladchikov, Y. Lithospheric scale folding: numerical modelling and application to the himalayan syntaxes. *International Journal of Earth Sciences* **88**, 190–200 (1999).

5. Hetényi, G. *et al.* Density distribution of the india plate beneath the tibetan plateau: Geophysical and petrological constraints on the kinetics of lower-crustal eclogitization. *Earth and Planetary Science Letters* **264**, 226–244 (2007).

6. Herman, F. *et al.* Exhumation, crustal deformation, and thermal structure of the nepal himalaya derived from the inversion of thermochronological and thermobarometric data and modeling of the topography. *Journal of Geophysical Research: Solid Earth* **115** (2010).

7. van Dinther, Y. *et al.* The seismic cycle at subduction thrusts: Insights from seismo-thermo-mechanical models. *Journal of Geophysical Research: Solid Earth* **118**, 6183–6202 (2013).

8. Turcotte, D. & Schubert, G. *Geodynamics* (Cambridge University Press, 2002).

9. Cattin, R. & Avouac, J. Modeling mountain building and the seismic cycle in the himalaya of nepal. *Journal of Geophysical Research* **105**, 13389–13407 (2000).

10. Bollinger, L., Avouac, J., Cattin, R. & Pandey, M. Stress buildup in the himalaya. *Journal of Geophysical Research: Solid Earth (1978–2012)* **109** (2004).

11. Gillespie, P., Walsh, J. t. & Watterson, J. Limitations of dimension and displacement data from single faults and the consequences for data analysis and interpretation. *Journal of Structural Geology* **14**, 1157–1172 (1992).

12. Hirose, T. & Bystricky, M. Extreme dynamic weakening of faults during dehydration by coseismic shear heating. *Geophysical Research Letters* **34** (2007).

13. Di Toro, G. *et al.* Fault lubrication during earthquakes. *Nature* **471**, 494–498 (2011).

14. Del Gaudio, P. *et al.* Frictional melting of peridotite and seismic slip. *Journal of Geophysical Research: Solid Earth* **114** (2009).

15. Elliott, J. *et al.* Himalayan megathrust geometry and relation to topography revealed by the gorkha earthquake. *Nature Geoscience* (2016).

16. Duputel, Z. *et al.* The 2015 gorkha earthquake: A large event illuminating the main himalayan thrust fault. *Geophysical Research Letters* **43**, 2517–2525 (2016).

17. Wang, Q. *et al.* Present-day crustal deformation in china constrained by global positioning system measurements. *science* **294**, 574–577 (2001).

18. Zhao, W. *et al.* Deep seismic reflection evidence for continental underthrusting beneath southern tibet. *Nature* **366**, 557 (1993).
