# Supplementary material for: Bimodal seismicity in the Himalaya controlled by fault friction and geometry
Source: Nat Commun. 2019 Jan 3;10:48. doi: 10.1038/s41467-018-07874-8 (PMC6318329; doi:10.1038/s41467-018-07874-8)
Supplement: Supplementary file 3 — Description of Additional Supplementary Files [file 41467_2018_7874_MOESM3_ESM.pdf]

Description of Additional Supplementary Files

Supplementary Movie 1

Description: Animation of the shear strain rate (ϵ_{xy}) showing a sequence of three rupture events, including two consecutive partial ruptures and a final complete rupture, which propagates throughout the seismogenic zone.
